# tRNA-Derived Small RNA: A Novel Regulatory Small Non-Coding RNA

**DOI:** 10.3390/genes9050246

**Published:** 2018-05-10

**Authors:** Siqi Li, Zhengping Xu, Jinghao Sheng

**Affiliations:** 1 Institute of Environmental Health, School of Public Health, Zhejiang University, Hangzhou 310058, China; lisiqi@zju.edu.cn (S.L.); zpxu@zju.edu.cn (Z.X.); 2 Collaborative Innovation Center for Diagnosis and Treatment of Infectious Diseases, Zhejiang University, Hangzhou 310003, China; 3 Program in Molecular and Cellular Biology, School of Medicine, Zhejiang University, Hangzhou 310058, China

**Keywords:** transfer RNA, transfer RNA-derived small RNA, small non-coding RNA, biological function

## Abstract

Deep analysis of next-generation sequencing data unveils numerous small non-coding RNAs with distinct functions. Recently, fragments derived from tRNA, named as tRNA-derived small RNA (tsRNA), have attracted broad attention. There are mainly two types of tsRNAs, including tRNA-derived stress-induced RNA (tiRNA) and tRNA-derived fragment (tRF), which differ in the cleavage position of the precursor or mature tRNA transcript. Emerging evidence has shown that tsRNAs are not merely tRNA degradation debris but have been recognized to play regulatory roles in many specific physiological and pathological processes. In this review, we summarize the biogeneses of various tsRNAs, present the emerging concepts regarding functions and mechanisms of action of tsRNAs, highlight the potential application of tsRNAs in human diseases, and put forward the current problems and future research directions.

## 1. Introduction

Transfer RNAs (tRNAs) are ubiquitous nucleic acid entities that belong to the most abundant small non-coding RNA, constituting 4–10% of all cellular RNA [1]. They are the fundamental components of the translation machinery in that they deliver amino acids to the ribosome to translate the genetic information in an mRNA template into a corresponding polypeptide chain. Traditionally, tRNA is considered as a static contributor to gene expression and its function is limited as an adaptor molecule in translation [2]. However, recent studies have revealed that a large number of non-coding small RNAs are derived from tRNAs. The tRNA-derived small RNAs (tsRNAs) are not merely byproducts of random cleavage of tRNAs, but as regulatory small non-coding RNAs in pathophysiologic processes [3]. Thus, tRNA has now been recognized as a dynamic regulator with various functions.

The biogenesis of mature tRNA begins with the transcription of precursor tRNA (pre-tRNA) by RNA polymerase III. The 5′ leader sequence and a 3′ polyuracil (poly-U) tail of a newly transcribed pre-tRNA can be cleaved by endonucleolytic ribonuclease P (RNase P) and ribonuclease Z (RNase Z), respectively, followed by the addition of a 3′ CCA tail with the help of the tRNA nucleotidyl transferase. For a subset of tRNAs, the intron located between position 37 and 38 are removed through tRNA splicing. Multiple post-transcriptional modifications are also added along the maturation of a tRNA. Only correctly processed tRNAs leave the nucleus through a nuclear receptor-mediated export process, which serves as a checkpoint for sorting tRNAs with incorrectly processed termini [4]. The mature tRNAs have a length of 73–90 nts and adopt a clover-leaf shaped secondary structure, composing a D-loop, an anticodon loop, a T-loop (also known as TψC loop), a variable loop, and an amino acid acceptor stem [1,5]. Since the beginning of this century, researchers have found that tRNAs can be precisely regulated and cleaved into tsRNAs, which play unique and diverse biological roles under different physiological and pathological conditions. Therefore, studying the biological functions and the underlying molecular mechanisms of tsRNAs has become a new research active spot in the field of small non-coding RNA. Here, we present updated views concerning the biogenesis, biological functions, and mechanisms of action of tsRNAs, and discuss their involvement and potential application in human health and diseases.

## 2. tRNA-Derived Small RNA Classification and Biogenesis

**Figure 1:**
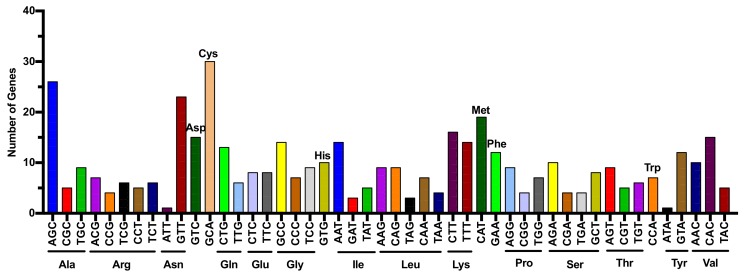
Multiple transfer RNA (tRNA) genes in human genome. Numbers of tRNA genes are presented for different amino acids and anticodons. Each graph with different colors indicates a different amino acid. Various isoacceptors (tRNA acceptors that accept the same amino acids) exist for each amino acid. Existence of isodeciders (tRNA genes with the same anticodon but different sequences elsewhere in the tRNA body) for each isoacceptor expands the source of RNAs for generation of diverse tRNA-derived small RNAs (tsRNAs).

The discovery of tsRNAs can be dated back to the late 1970s, when they were considered as product of tRNA random degradation and attracted only minimal interest [6,7]. The wide application of high-throughput sequencing technology has unveiled various tsRNAs in bacteria, fungi, plants, and mammals. tRNAs are encoded by multiple genes in human (Figure 1). Since there are multiple isoacceptors (tRNA acceptors that take the same amino acids) for each amino acid and numerous isodecoders (tRNA genes with the same anticodon but different sequences elsewhere in the tRNA body) for individual isoacceptor, various types of tsRNA can be generated from diverse tRNA sources. Based on the length and cleavage sites of tRNAs, tsRNAs can be divided into two main types: one is stress-induced tRNA fragments, produced by specific cleavage in the anticodon loop of mature tRNAs with 28–36 nts length, which has been named as tRNA-derived stress induced RNA (tiRNA); the other is about 14–30 nts length and derived from the mature or primary tRNAs, which has been named as tRNA-derived fragment (tRF). Both types of tsRNA can accumulate during different biological processes in several species and have very different biogenesis pathways that are slowly being uncovered. Moreover, the naming conventions of tsRNAs are inconsistent, as researchers generally named the identified tsRNAs in their systems primarily based on their own preferences. The un-unified tsRNA naming system indicates that the research in this field is not mature enough and has a broad research and development space. 

### 2.1. tRNA-Derived Stress-Induced RNA

**Figure 2:**
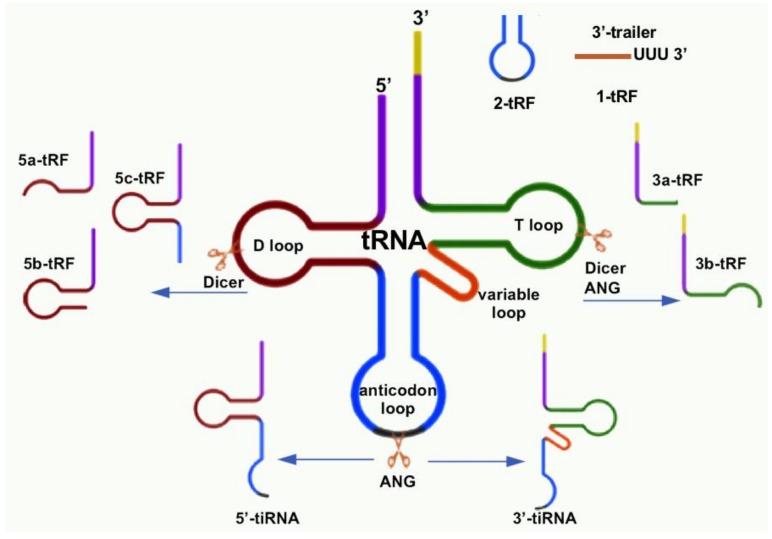
The types of tsRNAs are classified by size and sequence location in the tRNA structure. 1-tRNA-derived fragment (tRF) is generated by RNase Z, which cleaves 3′ trailer from pre-ribosomal RNA (rRNA). 2-tRF, which contains the anticodon loop, is generated by unknown ribonuclease. In the case of 3-tRFs and 5-tRFs, the subtypes are determined by size and location of the source. tiRNAs are grouped by whether their source sequences are from the 5′ or 3′ tRNA cleaved by angiogenin (ANG).

Two subtypes of tiRNAs have been discovered, namely 5′-tiRNAs and 3′-tiRNAs, which are generated by the cleavage in or near the anticodon loop of a tRNA (Figure 2). Rny1p, a member of the Ribonulcease T2 family, and angiogenin (ANG), a member of the Ribonuclease A superfamily, are responsible for the production of tiRNAs in yeast and mammal cells, respectively [8,9,10]. The cleavage events are executed under stress conditions such as amino acid deficiency, phosphate starvation, UV radiation, heat shock, hypoxia, oxidative damage, and viral infection [8,10,11,12,13,14,15]. Nonetheless, it has been found that very few tiRNAs are produced under growth condition [16]. tiRNAs are mainly located in the cytoplasm, with a small amount in the nucleus and mitochondria, and can also be detected in the circulation system of human [17,18].

### 2.2. tRNA-Derived Fragment

tRFs are produced from pre-tRNAs as well as mature tRNAs. Up until now, four types of tRFs have been identified and characterized by their provenance on tRNAs: 5-tRFs, 3-tRFs, 1-tRFs, and 2-tRFs (Figure 2). 5-tRFs are generated from cleavage in the D-loop of tRNAs by Dicer, oftentimes with adenine as their 3′-ends, and can be further divided into 5a-tRFs (~15 nts), 5b-tRFs (~22 nts), and 5c-tRFs (~30 nts) [19,20]. 3-tRFs are resulted from cleavage in the T-loop by Dicer, ANG or other members of the Ribonuclease A superfamily, and usually contain a CCA tail sequence with a length of about 18 nts or 22 nts [19,20,21,22]. Cleavage of the 3′-trailer fragment of pre-tRNAs by RNase Z or its cytoplasmic homologue ELAC2 give rise to 1-tRFs, which usually begin exactly after the 3′-ends of mature tRNA (5′-CCA-3′ tails excluded) and possess poly-U at their 3′-ends [20,23,24]. Recently, a novel tRF subtype containing the anticodon loop has been detected in breast cancer cells. They are derived from tRNA^Glu^, tRNA^Asp^, tRNA^Gly^, and tRNA^Tyr^, and are referred as 2-tRFs. However, the ribonuclease responsible for the production of 2-tRFs is still not clear [25]. The subcellular localization of tRFs is still controversial, partially due to the variations in experimental approaches used and species. For examples, in human cells, 5-tRFs are abundant in nucleus while 3-tRFs and 1-tRFs are in cytoplasm [26,27,28]; however, in *Drosophila* and *Trypanosoma cruzi* cells, tRFs were found to be exclusively cytoplasmic [29,30]. The specific subcellular localization of tRFs also depends on how they are generated and is believed to be important for their differential cellular functions.

### 2.3. Effects of Transfer RNA Modification and Structure on tRNA-Derived Small RNA Biogenesis

In eukaryotes, tRNAs undergo by far the most numerous and chemically diverse post-transcriptional modifications that ensure proper structure and function. The prevalence of modified nucleotides in tRNAs varies in different organisms, with the density of nucleotide modifications making up about 17% of the total residues [31]. Most of these modifications are located in the anticodon loop, with methylation at position 34 (the first one of anticodon) and 38 (close to the 3′ end of the anticodon) affecting tRNA function in translation and increasing the accuracy of translation [32]. It has also been revealed that the tRNA modifications could influence the biogenesis of tsRNAs. For example, two methyltransferases NSun2 and Dnmt2, can promote tRNA stability by specifically methylating C5 at cytosine residues (m^5^C) [33]. Thus, in NSun2 knockout mice that fail to implement tsRNA m^5^C modifications, the cleavage of tsRNAs into tiRNAs by ANG is greatly increased, leading to repression of translation, induction of a stress response and subsequent apoptosis in cerebral cortex, hippocampus and striatum neurons [17,34]. Similarly, Dnmt2 mediated m^5^C modifications inhibit the ANG mediated cleavage of tRNA anticodon loop, resulting in decreased levels of tiRNAs [33,35]. In addition, the tRNA tertiary conformation is also essential for preventing the cleavage of tRNA by ANG. A recent report has shown that stress conditions such as irradiation, toxicity, ischemia, and acute injuries can disrupt the tertiary structures of mature tRNAs, enabling the tRNAs to be cleaved by ANG to play a protective role [36]. Therefore, the modifications and conformations of mature tRNAs are necessary for the maintenance of tRNA structure and the blockage of endonuclease cleavage, suggesting tsRNAs (especially tiRNA) are likely derived from tRNAs that are not properly modified or folded.

## 3. Biological Roles and Mechanisms of Action of tRNA-Derived Small RNAs

**Figure 3:**
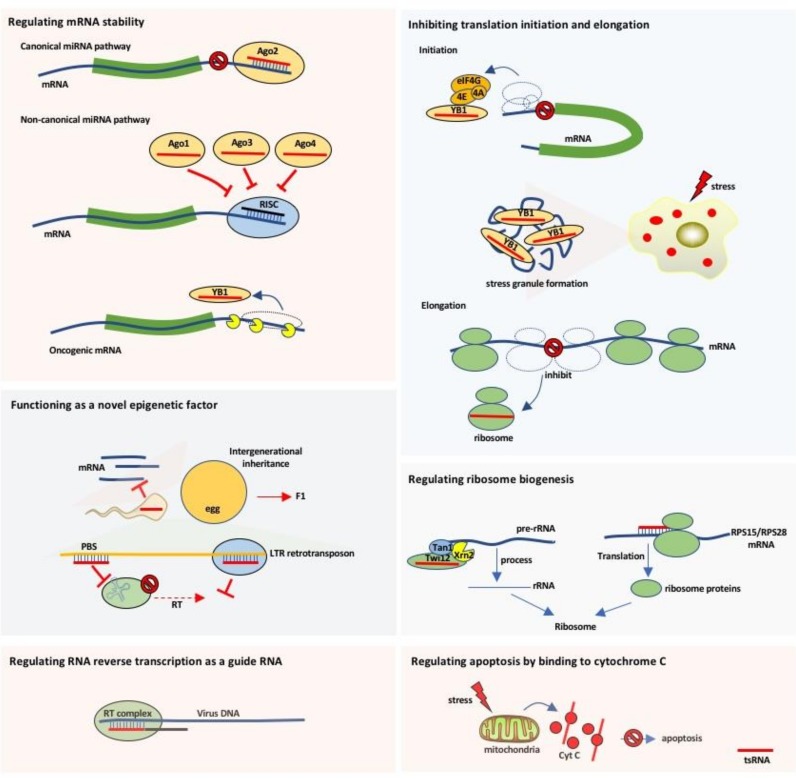
The diverse functions and working mechanisms of tsRNAs. It is generally accepted that the tsRNAs regulate a variety of biological processes, including gene expression, translation initiation and elongation, stress granule assembly, ribosome biogenesis, intergenerational inheritance, and apoptosis. miRNA: microRNA; RT: reverse transcription; LTR: long terminal repeat; RISC: RNA-induced silencing complex; PBS: primer binding sites; YB1: Y-box binding protein 1.

Despite mounting evidence for the generation of tsRNAs in almost all cell types, a recurring concern is that tsRNAs might be aberrant degradation products of certain endonucleases. Numerous groups have demonstrated that the processing of mature tRNAs into tsRNAs is remarkably site-specific and restricts to some isotypes of a tRNA. More importantly, tsRNAs exhibit features of functional regulatory small non-coding RNAs, which are shown in Figure 3.

### 3.1. Regulating mRNA Stability

As a kind of small non-coding RNAs with length less than 30 nts, tRFs hold similar functions with microRNAs (miRNAs) which regulate mRNA stability by directly binding to mRNA. For example, 3-tRFs derived from tRNA^Gly-GCC^ in mature B lymphocytes (referred as CU1276) or from tRNA^Leu-CAG^ in non-small cell lung cancer cells have has miRNA-like structure and function, inhibiting protein translation or cleaving partially complementary target site [37,38]. Since tRFs are evolutionarily ancient and present in both prokaryotes and eukaryotes [27], it is not surprising that tRFs in *Drosophila* preferentially suppress mRNA translation of ribosomal proteins and other translational factors via antisense pairing [39]. Interestingly, some tsRNAs preferentially associate with Ago1, Ago3, and Ago4 proteins but not Ago2 in a cell type specific manner [27], indicating that tRFs have other functions than the direct binding with their target genes as miRNAs. Haussecker et al. reported that 3-tRFs and 1-tRFs can regulate the gene expression by competitively binding to Ago family proteins, thus affecting the silence efficiency of their target genes [28]. For example, 1-tRF^Ser-TGA^ derived from pre-tRNA^Ser-TGA^ (referred as Cand45) interacts with Ago3 and Ago4 but does not have a gene silencing function as seen in miRNAs [28]. Therefore, tRFs could regulate gene expression through either canonical or non-canonical miRNA/siRNA pathway by incorporating into Ago proteins. Since the Ago proteins bind small RNAs ranging from 20 to 24 nts [40], tiRNAs may not be incorporated into these proteins and exert their functions through miRNA/siRNA pathways. In addition, 2-tRFs (mainly from tRNA^Glu^, tRNA^Asp^, tRNA^Gly^, and tRNA^Tyr^) can competitively bind to YBX-1 (also referred as YB-1) and block its interaction with oncogenic mRNAs, thereby reducing the stability of these mRNAs and eventually inhibiting metastasis of human breast cancer cells [25]. Further efforts are needed to explore the endogenous mRNA targets of tRFs and to construct the regulatory networks.

### 3.2. Inhibiting Translation Initiation and Elongation

The tiRNAs (mainly 5′-tiRNAs) could decrease the global translation speed by 10–15% [10]. Since tRNAs are key molecules of protein biosynthesis, inhibition of translation as a consequence of full length tRNA destruction seems to be a plausible scenario. However, this picture appears to be too simplistic, as it has been shown in several studies that the steady state levels of genuine tRNAs do not change significantly [10,14,41]. Subsequent studies showed that specific tiRNAs (mainly 5′-tiRNAs from tRNA^Ala^ and tRNA^Cys^) could assemble into a G-quadruplex-like structure (G4-motif), which is able to competitively bind to eIF4G/eIF4A in the translation initiation complex and then inhibit cap-dependent mRNA translation but not the translation of internal ribosome entry sites (IRES)-mediated protein translations which are responsible for cell survival and anti-apoptosis [42,43]. Given that most proteins responsible for cell survival and anti-apoptosis are translated via the IRES pathway, it is likely that tiRNAs can exert protective effects under stress conditions by selectively repressing the translation of housekeeping components to reduce cellular energy expenditure while leaving the production of pro-survival proteins unaffected. These findings indicate that the purpose of tiRNA production is not to decrease the functional mature tRNA level but to regulate translational process under stress conditions. In addition, 5′-tiRNAs can also bind to the translation silencer YB-1 and enhance the assembly of stress granule through an eIF2α phosphorylation independent pathway [44], so as to facilitate cellular resistance to stress and promote survival. We therefore believe that tiRNAs represent an important regulatory factor under stress.

A recent study unveils that pseudo-uridylation of 5-tRFs can also steer translation initiation in stem cells [45]. However, several lines of experimental evidence indicate that tRFs affect the elongation phase of translation. tRFs may serve as molecular brakes for polysome assembly with consequent translational repression [46]. For example, 5-tRFs are able to repress the expression of reporter genes without the needs for complementary sites in the mRNA, implicating tRFs may target the translational machinery [47]. A 5-tRF derived from tRNA^Val-GAC^ in *Haloferax volcanii* has been demonstrated to bind the small ribosomal subunit in immediate vicinity of the mRNA channel and dim protein biosynthesis globally [48]. The detailed structural studies may be required to better understand how tRFs associate with ribosome components. Taken together, it appears reasonable to assume that tsRNAs can attenuate global protein biosynthesis by targeting the translation machinery.

### 3.3. Regulating Ribosome Biogenesis

tsRNAs have recently emerged as important regulators of ribosome biogenesis, including ribosomal RNAs (rRNA) and proteins. In lower organisms *Tetrahymena thermophila*, it is found that tsRNA is a composition of the pre-rRNA splicing complex (TXT) [49]. 3-tRFs can specifically bind to Twi12 protein (an Ago/Piwi family protein), thus recruits Tan1 protein and exonuclease Xrn2 to form a TXT complex, which cleaves and processes the precursor rRNAs to enhance rRNA synthesis. However, whether tRFs could enhance rRNA process in higher organisms needs further investigation. In mammalian cells, 3-tRF derived from tRNA^Leu-CAG^ can bind at least two ribosomal protein mRNAs (ribosomal protein S28, RPS28 and ribosomal protein S15, RPS15) to promote their translations [50]. However, there is no direct evidence showing tiRNAs are able to regulate ribosome biogenesis. For now at least, tsRNAs modulate translation at different levels depending on their subtypes, the cellular states, and species.

### 3.4. Functioning as a Novel Epigenetic Factor

Transposable elements are mobile genomic DNAs that are potentially harmful to the host genome, and their transcription is normally suppressed by epigenetic marks such as DNA methylation and histone modification [51]. However, it remains an open question how the genome defends itself during the window of epigenetic reprogramming, such as pre-implantation embryo development, when most of the epigenetic marks are wiped off and then re-established. Recently, Andrea et al. discovered that 3′-tRFs with different lengths (18 nts and 22 nts) can silence the long terminal repeat (LTR) retrotransposon by blocking reverse transcription (the 18 nts 3-tRF) and miRNA-like post-transcriptional silencing (the 22 nt 3-tRF), respectively [52]. In plant *Arabidopsis thaliana*, 5-tRFs are processed by Dicer-like 1 and incorporated into Ago1, akin to a miRNA involved in the regulation of genome stability through targeting to transposon element transcripts [53]. Interestingly, tiRNAs are the most abundant small RNA species in the mature sperm of mice [54]. The sperm tiRNAs delivered at fertilization can change the transcriptome of the early-stage embryos, including genes associated with metabolic pathways [55,56]. These changes are not associated with the DNA methylation status, suggesting that the regulatory sperm tiRNAs may act as a novel epigenetic factor that affects the phenotype of the offspring. These works open yet another chapter about the RNA mediated epigenetic control, but the precise contribution of tsRNAs in vivo might be more complicated and deserves case-by-case study.

### 3.5. Regulating RNA Reverse Transcription as a Guide RNA

tsRNAs can be used as a guide RNA in virus RNA reverse transcription. The 3-tRF (referred as tRF-3019) of host cells could bind to the primer-binding sites (PBS) of human T-cell leukemia virus type 1 (HTLV-1) RNA, thus initiating reverse transcription and promoting the virus self-synthesis [57]. Meanwhile, respiratory syncytial virus (RSV) infection can trigger stress response in the host cells through inducing ANG cleavage of tRNAs to produce tiRNAs. RSV utilizes the host tiRNAs as primers to favor its replication and improve the infection efficiency [58,59,60]. Thus, both two types of tsRNA can serve as primers for reverse transcription. Further studies on the functions of tsRNAs in viral infection are likely to provide valuable insights into details of virus–host interactions.

### 3.6. Preventing Apoptosis by Binding to Cytochrome C

An early study has shown that mature tRNA molecules could bind to Cytochrome C (Cyt C), and then inhibit the apoptosome formation and the activity of Caspase 9, thus promoting cell survival [61]. As tsRNAs are derived from tRNAs, it is speculated that tsRNAs may also interact with Cyt C. Under hyperosmotic stress condition, ANG mediated tiRNAs (both the 5′-tiRNA and 3′-tiRNA) but not fully tRNAs or tRFs can interact with mitochondria-released Cyt C to form ribonucleoprotein complex, thus inhibiting the apoptosome formation and activity [62]. The interaction between tiRNAs and Cyt C triggers a series of biological processes leading to suppression of apoptosis, and this is gradually considered as a new anti-apoptotic mechanism. However, how can tiRNAs specifically recognize Cyt C in this biological process? Whether its own modifications or some other factors are involved? Do the tRFs interact Cyt C in specific conditions? All these interesting questions need to be further investigated.

### 3.7. Immune Regulation

The tRFs exist in both the hematopoietic and lymphoid tissues and in blood circulation system. During the acute inflammation stage, the levels of tRFs rapidly increase in the circulatory system [18,63], indicating that tRFs likely probably play important roles in the immune response. On one hand, tRFs are involved in the gene regulation within immune cells. During the maturation of monocytes to dendritic cells, the 5-tRF derived from tRNA^Glu^ can form a complex with Ago-like proteins PIWIL4 and PIWIL1, and then recruits SETDB1, SUV39H1, and HP1beta to methylate the histone H3K9 on the promoter region of CD1A, leading to inhibition of CD1A expression [64]. On the other hand, tsRNAs (e.g., CCACCA sequences at 3′-end of tRNA-AlaUGC) can directly interact with Toll-like receptors to activate the immune responses of Th1 and toxic T lymphocytes [65]. These results suggest that tsRNA is a novel immune signaling molecule, but the underlying mechanisms need to be further explored.

### 3.8. Other Possible Roles and Mechanisms of Action

tRNAs could also fold into stem-loop hairpin structures rather than the canonical tRNA clover-leaf structure to serve as conventional pre-miRNA, which then generates miRNA-like tsRNAs to silence certain mRNAs [66]. tsRNAs have also been found to exist in processing bodies (P-bodies, i.e., the degradation region of mRNA), indicating that tsRNAs may be directly involved in the degradation of target mRNAs like miRNAs [67]. Although some isolated functions have been reported, the vast majority of tsRNAs appear to operate via unknown mechanisms.

## 4. Roles of tRNA-Derived Small RNA in Diseases

Abnormal levels of tsRNAs have been observed in a variety of human diseases, including cancer, neurodegenerative diseases, acquired metabolic diseases, and infectious diseases. It remains to be determined whether these tsRNAs contribute to disease pathogenesis. Their involvements in human diseases provide fresh perspectives for the exploration and development of new biomarkers and novel therapeutic strategies for the detection, monitoring, and treatment of human diseases.

### 4.1. tRNA-Derived Small RNA and Cancer

Rapidly growing tumor cells outgrow a deficient blood supply which results in a microenvironment with limited oxygen and nutrients. Tumor cells can adapt to this stressful environment by different regulation strategies, thus ensuring survival and proliferation [68]. The tsRNA production from tRNAs under stress is one of the important pathways; accordingly, the biological functions of tsRNAs are mainly to protect cell survival under stress. Therefore, it is reasonable to believe that tsRNA is a new target for tumor therapy. Besides, tsRNAs can be detected in the urine and serum from tumor patients [69,70,71], suggesting they can be used as molecular markers for tumor diagnosis. For example, Dhahbi et al. found that the level of tsRNAs in the blood samples from breast cancer patients was closely related to the pathological characteristics through small RNA high-throughput sequencing analysis [72]; Honda et al. found that a kind of hormone dependent tsRNA was abundant in breast cancer and prostate cancer and can enhance the proliferation of cancer cells [16]. Therefore, a tsRNA database related to different tumors was established for researchers to query (tRF2Cancer) [73]. Moreover, the presence of mutations at the tsRNA (Reference to ts-101/ts-53) gene locus in chronic lymphocytic leukemia (CLL) and lung cancer samples supports the idea that these tsRNAs can represent a new diagnostic marker in cancer management [74,75]. In summary, tsRNAs are closely related to tumor onset, progression, and drug response, and could be potential therapeutic targets and/or diagnostic markers; however, thorough and deliberate evaluations are needed before translating tsRNAs into clinics.

### 4.2. tRNA-Derived Small RNA and Neurodegenerative Diseases

It is generally believed that neurodegenerative diseases are mainly caused by genetic and environmental factors which induce oxidative stress, inflammation, and mitochondrial dysfunction, leading to neuron dysfunction. In recent years, a number of mutations were found in genes associated with tsRNA biogenesis from patients with neurodegenerative diseases, providing a basis for further investigations to link tsRNAs with the development of neurodegeneration [17,35,76,77].

Since 2004, more than 40 ANG mutants have been found in amyotrophic lateral sclerosis, Parkinson’s disease and other neurodegenerative diseases. Most of these mutations impair its ribonucleolytic activity or/and nuclear translocation [76]. Considering that the biogenesis of many tiRNAs depends on the ribonucleolytic activity of ANG, it is reasonable to speculate that certain tiRNAs play important roles in these diseases. In fact, it has been shown that tiRNAs (such as tiRNA^Ala^ and tiRNA^Cys^) or their DNA analog with G4-motif could promote neuron survival under stress conditions and could be used for the treatment of neurodegenerative diseases [42,78]. Thus, individuals with ANG mutations are more likely to succumb to neurodegeneration. On the contrary, there are reports indicating that ANG can induce accumulation of tiRNAs caused by defects in tRNA methyltransferases Dnmt2 and NSun2, thus triggering a stress response and cell death in the nervous system, suggesting tiRNAs enhance neuron death [17,35]. Therefore, tiRNAs seem to have opposite effects, either protecting the neurons or promoting neuronal damage. We believe that the levels and types of tiRNAs are the key points to determine their roles in the process of cell damage: at the early stage, the production of tiRNAs will reduce the protein translation speed and activate a stress response, protecting cell survival; however, if the cell cannot be recovered, the existence of tiRNAs will lead to sustained stress, eventually damaging the cells. Although the true functions of different tsRNAs in neurodegeneration are still unclear, these studies indicate that there is a close relationship between abnormal tRNA metabolism and neurodegenerative diseases [77].

### 4.3. tRNA-Derived Small RNA and Acquired Metabolic Diseases

In 2012, a novel class of tsRNA, which is derived from the 5′-end of tRNAs and ranged from 30 to 34 nts in length, was first discovered in the mature sperm of mammals [54]. Although details of their biogeneses remain unknown, sperms show an altered tsRNA profile after a high-fat diet (HFD) [55] or a low-protein diet in mice [79], after an HFD [80] or an environmental-like compound exposure in rats [81], and in obese human [82], suggesting that sperm tsRNA may be functionally involved in acquired metabolic diseases. Functional evidence provides that a tsRNA-dependent mechanism mediates the paternal transmission of metabolic traits in mammals [55]. Subsequent studies showed that significant changes in pancreatic islet transcriptome (mainly metabolism related pathways) in the early-stage embryos injected with sperm tsRNAs from high-fat mice and in the offspring mice [55,56]. Moreover, sperm tsRNAs were found to harbor numerous RNA modifications that contribute to the stability of tsRNAs, and levels of 5-methylcytidine (m^5^C) and N_2_-methylguanosine (m^2^G) in sperm tsRNAs were significantly increased after paternal HFD consumption [55]. These discoveries raise exciting new possibilities regarding the mechanisms through which sperm gain information from the environment through tsRNAs mediated epigenetic memory, which contributes to the intergenerational inheritance of an acquired metabolic disorder.

### 4.4. tRNA-Derived Small RNA and Infectious Diseases

tsRNAs were observed in some types of infectious agents, such as *Escherichia coli* [83], *Aspergillus fumigatus* [12], *Giardia lamblia* [13], *Ascaris* [84], *Trypanosoma cruzi* [85,86], viruses [58,59,60], and prions [87], under different stimuli that could be stress, cell cycle progression, and/or infection by a pathogenic agent [88]. For example, *T. cruzi* secreted a population of small non-coding RNAs including tsRNAs in extracellular vesicles and transferred to other parasites and susceptible mammalian cells, leading to metacyclogenesis transformation and to an increased susceptibility to infection [86]. However, the consequences or biological roles of the tsRNAs in infectious agents are not completely defined. Further efforts should be focused on the complete description of tsRNAs and their roles as virulence factors, drug targets, biomarkers, or as relevant molecules for infection.

4.5. tRNA-Derived Small RNA as a Biormarker for Disease Diagnosis

Study of the association between the circulating tsRNA level and disease represents an active spot in this field. RNA high-throughput sequencing analysis revealed that tsRNAs are significantly different in urine and serum between cancer patients and healthy people and are associated with the pathological processes of cancer [72]. Meanwhile, it was found that the tsRNA content in serum is closely related to aging, calorie intake, and acute tissue injury [18,36]. Moreover, the levels of tsRNAs in tissue section can also be used as a marker for disease severity. For example, 2-tRFs containing anticodon loop are accumulated in primary tumor [25]; tsRNAs content in tissue is correlated to the clinical pathological characteristics of breast cancer [72], and is significantly increased in damaged tissues [36]. However, the study on tsRNAs as a biomarker for disease diagnosis is still at a preliminary stage, and more clinical and experimental evidence is needed for clinical translation.

## 5. Conclusions and Perspectives

In summary, the research on tsRNAs has made some important progress and become a new hot spot in the field of non-coding RNAs. It has been found that tsRNAs play different roles in gene expression, protein translation, epigenetic regulation, and immune processes, and are closely associated with various diseases such as cancer, neurodegenerative diseases, metabolic diseases, and infectious diseases. However, the study on tsRNAs is still at a preliminary stage, and there are many questions that need to be solved.

Firstly, the biogenesis process of tsRNAs is not completed understood. Although it is known that RNase Z, Dicer, and ANG are involved in the biogeneses of tsRNAs, the understanding of ribonucleases is not very comprehensive. For example, the ribonuclease responsible for the biogenesis of 2-tRF is still not identified. Meanwhile, it is not clear about the underlying molecular mechanism of tsRNA biogenesis. It is necessary to screen and identify the relevant regulatory factors, and observe the dynamic processes in cells. In addition, as a new non-coding small RNA, are tsRNAs stable in cells? Is there any modification? How they are degraded? All these questions need to be further studied.

Secondly, the naming of tsRNA is still inconsistent. The human genome contains more than 500 tRNA genes, and all the tRNAs could be cleaved by different types of ribonucleases to produce various tsRNAs. However, these tsRNAs have not been categorized with a unified name yet. Although some tsRNA databases have simply classified and encoded some tRFs according to the naming mode of miRNA, a large number of tsRNAs have been missed and the simple classification and encoding mode cannot provide clear basic information about the tsRNAs [19]. According to the origins and types of tsRNAs, we proposed a naming convention in the form of X-tsRNA^AA-NNN^. Here, tsRNA represents the species, and it is divided into tiRNAs and tRFs; X represents the subtypes of tsRNAs, which can be divided into 1, 2, 3, and 5 based on the mapped location of tRNAs; AA represents the abbreviation of amino acid carried by the mapped tRNAs; NNN represents the anticodon of the mapped tRNAs. For example, 5′-tiRNA and 3a-tRF derived from tRNA^Glu-CTC^ can be named as 5′-tiRNA^Glu-CTC^ and 3a-tRF^Glu-CTC^, respectively. The names of tsRNAs from published in the related articles can be re-named in Table 1.

Thirdly, the universality and specificity of tsRNAs are unclear. Our current understanding of the mechanism of tsRNAs is mainly restricted to a few specific tsRNAs. There are large types and numbers of tsRNAs. Therefore, is there any tissue specificity in their expression and distribution? Do their biological functions have universality or specificity? All these questions are still unclear. Furthermore, our experimental approaches and methods for tsRNAs are also limited. As tsRNAs are derived from tRNAs, it is still difficult to specifically change the tsRNAs content without affecting the expression level of mature tRNAs.

Finally, the clinical translation of tsRNAs needs in-depth evaluation. tsRNAs have been shown to be abnormally expressed in various diseases, such as cancer, neurodegenerative diseases, and metabolic diseases, underscoring their potential values in clinical application. It is found that during acute injury of tissues and organs, the tsRNAs level in the circulatory system is increased, making tsRNAs more sensitive than other known tissue injury markers. It is expected that tsRNAs can serve as novel markers for identifying organ damage [36]. However, current methods for detecting tsRNAs are mainly based on high-throughput sequencing and Northern blot, which are not suitable for clinical analysis of large samples. Therefore, the development of appropriate detection methods and the establishment of clinical indicators are the key steps for tsRNA to be applied clinically.

tsRNA, as a novel regulatory non-coding small RNA, not only broadens the research field of small non-coding RNA, but also enriches the content of tRNA as a dynamic factor involved. Even though our current knowledge about tsRNAs is yet incomplete, they clearly represent a novel class of regulatory small non-coding RNA with surprising cellular roles in all domains of life. Future work will reveal how they fit into the ever-growing puzzle of RNA biology.

## Figures and Tables

**Table 1:** The published names and suggested renaming of tsRNA.

tsRNA Type	Subtype	Published Name	Suggested Rename	Biogenesis	Refs.
tRF	1-tRF	tRF-1001	1-tRF^Ser-TGA^	pre-tRNA^Ser-TGA^	[20]
		Cand45	1-tRF^Ser-TGA^	pre-tRNA^Ser-TGA^	[28]
	2-tRF	tRF^Asp^, tRF^Glu^, tRF^Gly^	2-tRF^Asp-GTC^, 2-tRF^Glu-YTC^, 2-tRF^Gly-TCC^	tRNA^Asp-GTC^ tRNA^Glu-YTC^ tRNA^Gly-TCC^	[25]
	3-tRF	tRF-3019	3a-tRF^Pro^	tRNA^Pro^	[57]
		miR-1280	3a-tRF^Leu^	tRNA^Leu^	[66]
		miR-720	3a-tRF^Thr^	tRNA^Thr^	[66]
		miR-1274a/b	3a-tRF^Lys5^, 3a-tRF^Lys3^	tRNA^Lys^	[66]
		CU1276	3b-tRF^Gly-GCC^	tRNA^Gly-GCC^	[37]
	5-tRF	miR-1308	5a-tRF^Gly^	tRNA^Gly^	[66]
		miR-886-5P	5a-tRF^Ala^	tRNA^Ala^	[66]
		Val-tRF	5b-tRF^Val-GAC^	tRNA^Val-GAC^	[89]
		td-piR(Glu)	5c-tRF^Glu^	tRNA^Glu^	[64]
tiRNA	5′-tiRNA 3′-tiRNA	tRF5-GluCTC	5′tiRNA^Glu-CTC^	tRNA^Glu-CTC^	[58,59]
		tsRNA	e.g., 5′-tiRNA^Glu-CTC^, 3′-tiRNA^Glu-CTC^	tRNA	[55]
		SHOT-RNA	e.g., 5′-tiRNA^Glu-CTC^, 3′-tiRNA^Glu-CTC^	tRNA	[16]
		tRNA halve	e.g., 5′-tiRNA^Glu-CTC^, 3′-tiRNA^Glu-CTC^	tRNA	[12,90,91]
		tiRNA	e.g., 5′-tiRNA^Glu-CTC^, 3′-tiRNA^Glu-CTC^	tRNA	[42,43]

tiRNA: tRNA-derived stress-induced RNA; miR: microRNA; SHOT-RNA: sex hormone-dependent tRNA-derived RNA; piR: piwi-interacting RNA; td-piR: tRNA-derived piR.
